# Sterilization Induced Changes in Polypropylene-Based Ffp2 Masks

**DOI:** 10.3390/polym13234107

**Published:** 2021-11-25

**Authors:** Emmanuel Richaud, Muriel Ferry, Floriane Carpentier, Sophie Rouif, Laurent Cortella, Stéphane Esnouf

**Affiliations:** 1 Laboratoire PIMM, Arts et Metiers Institute of Technology, CNRS, CNAM, HESAM Université, 151 bd de l’Hopital, 75013 Paris, France; 2 CEA, Service d’Etude du Comportement des Radionucléides, Université Paris-Saclay, 91191 Gif-sur-Yvette, France; MURIEL.FERRY@cea.fr (M.F.); Floriane.CARPENTIER@cea.fr (F.C.); Stephane.ESNOUF@cea.fr (S.E.); 3 Ionisos SAS, 13 Chemin du Pontet, 69380 Civrieux-d’Azergues, France; Sophie.Rouif@ionisos.com; 4 ArcNucleart, CEA Grenoble, 17 Avenue des Martyrs, CEDEX 9, 38054 Grenoble, France; laurent.cortella@cea.fr

**Keywords:** polypropylene, FFP2 masks, sterilization, chain scissions, crosslinking

## Abstract

In the context of the SARS-CoV2 pandemic and because of the surgical and FFP2 mask (equivalent to the American N95 masks) shortages, studies on efficient sterilization protocols were initiated. As sterilization using irradiation is commonly used in the medical field, this method was among those that were evaluated. In this work, we tested irradiation under vacuum and under air (under both γ-rays and e-beams), but also, for acceptance purposes, undertook washing prior to the e-beam irradiation sterilization process. This article deals with the modifications induced by the sterilization processes at the molecular and the macromolecular scales on an FFP2 mask. Fourier transform infrared spectroscopy in attenuated total reflectance mode, size-exclusion chromatography and thermal-desorption–gas chromatography–mass spectrometry were used to characterize possible damage to the materials. It appeared that the modifications induced by the different sterilization processes under vacuum were relatively tenuous and became more significant when irradiation was performed using γ-rays under air.

## 1. Introduction

Due to the COVID-19 pandemic, an unexpected and huge quantity of surgical and FFP2 masks was needed in a very urgent timeframe, and mask production turned out to be overstretched by these events. For this reason, numerous task forces [1,2,3,4] were looking to find a way to sterilize used masks to be able to reuse them. Since sterilization is usually performed in the medical field using either γ-rays [5,6] or e-beam [7] irradiation, it initially seemed very interesting to sterilize masks using this protocol. Moreover, for acceptance purposes, washing prior to the sterilization process was found to be unavoidable and has already been addressed in recent papers [8]. However, it is well-known that irradiation induces changes in organic materials at the molecular, macromolecular and microscopic levels. Due to surgical and FFP2 masks being made of different layers of polypropylene, it was essential to ascertain the materials’ resistance to possible sterilization protocols, since PP is actually well known to be quite sensitive to radiolytic degradation [9,10]. Answers to this question arose during each previous pandemic—the last one being the Influenza one in 2009 [11,12,13]—but fell into disuse either when the pandemic was disappearing or when the mask production reached a sufficient level. Hence, this question must be addressed either for knowledge transfer purposes or for ecological reasons.

The filtration performances of masks first depend on their electrostatic properties together with their mechanical resistance. The filtration aspect was recently addressed in a previous paper [14], but the macromolecular aspects were less covered. Thus, we decided to pay attention to this second aspect through a screening of sterilization treatment on polymer architecture. Since the plasticity in PP was shown to be closely linked to residual molar mass values and crystallinity [15], we decided to focus on the modifications induced by the sterilization treatments at the macromolecular scale, together with a screening on the possible structural changes at the molecular scale, particularly dealing with the irradiation effect, due to this being less covered in existing reviews [16].

## 2. Experimental Section

### 2.1. Materials

**Figure 1:**
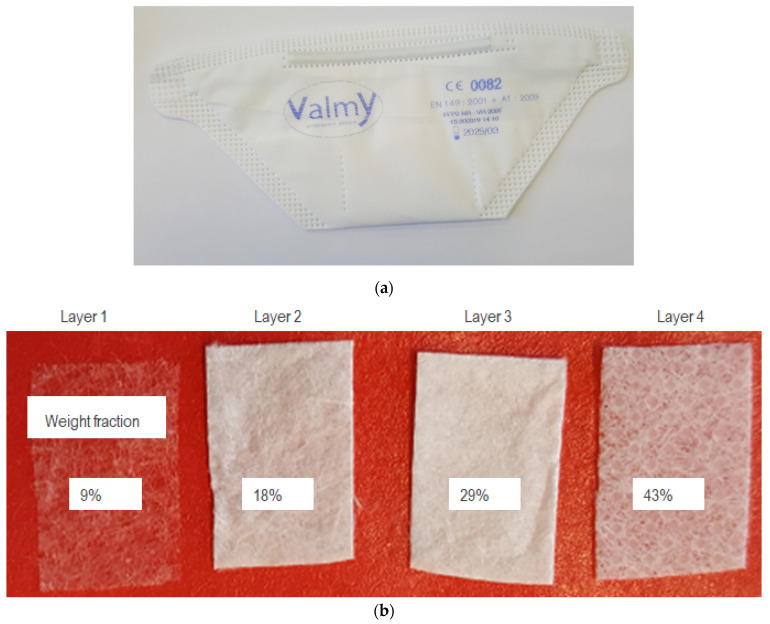
Picture of the Valmy FFP2 medical mask (**a**). The four layers of material (Layer 1: internal face; Middle: filter; Layer 4: external face). (**b**) The data for elastic strap are not presented in the main body of the article but its characterization was realized (see Figures S1–S4 of the Supplementary Materials).

Figure 1a presents a picture of the FFP2 medical mask manufactured by Valmy (Mably, France) and kindly supplied by Grenoble Hospital (see previous work [8]). Those masks are composed of four layers, which will be characterized before and after irradiation (Figure 1b).

### 2.2. Sterilization Protocols

In this work, various sterilization protocols were tested. They are summarized in Table 1. In case of the γ-ray irradiation performed at ArcNucleart, the dose rate was about 1.0 kGy h^−1^, whereas in the case of the 10 MeV e-beam irradiation performed at IONISOS (Chaumesnil, France) using a Mevex A29 device (34 kW, 10 MeV), the dose rate was several hundreds of kGy min^−1^.

When applicable, the washing conditions were as follows [8]: 1 h and 12 min at a constant temperature (60 °C), using either pure water or neutral detergent, with “Ultimate mineral” surfactants (1 mL/kg) added, along with “Ultimate Forte” disinfectant based on perchloric acid and hydrogen peroxide (5 mL/kg). Ultimate detergent was chosen because it is commonly used in hospitals.

When applicable, vacuuming was performed with a vacuum sealer, Model G210, with vacuum seal rolls from the same supplier (KitchenBoss, Shenzhen, China).

### 2.3. Characterizations

#### 2.3.1. Fourier Transform-Infrared Spectroscopy (FTIR)

Fourier Transform InfraRed spectra of the polymers were acquired using a Bruker Vertex 70 spectrometer equipped with a Specac Golden Gate single reflection diamond attenuated total reflectance (ATR) accessory and a DTGS (Deuterated TriGlycine Sulfate) detector. Acquisition was performed between 4000 and 500 cm^−1^ by adding 64 scans with a 4 cm^−1^ resolution. Although being a non-quantitative method, this technique was chosen due to its ease of use and lack of need for sample preparation, together with its ability to give significant information on the molecular and morphological changes.

#### 2.3.2. Size Exclusion Chromatography (SEC)

Size-exclusion chromatography (SEC) measurements were performed using a high-temperature gel permeation chromatograph (PL-GPC 220^®^ Agilent Technologies, Santa Clara, CA, USA). The samples were dissolved with 1,2,4-trichlorobenzene (Chromasolv, Sigma–Aldrich^®^) at a concentration of 1 mg∙mL^−1^. The eluent contained 0.03 wt% of 2,6-di-tert-butyl-4-methylphenol (BHT, Fluka, Illkirch, France) to stabilize the polymer against oxidative degradation. The solutions were stirred during 3 min at 135 °C and filtered before injection. The separation was carried out using two PLGel Olexis^®^ columns protected by a guard column. Peaks were detected with a refractive index detector (Agilent, Saint-Quentin-Fallavier, France). The temperature was set at 135 °C, and the flow rate was 1 mg∙mL^−1^. The calibration curve was established with the help of 4 Polystyrene Shodex^®^ (Tokyo, Japan) narrow standards of the respective molecular weights of 1.47 × 10^6^, 2.57 × 10^5^, 4.65 × 10^4^ and 7.21 × 10^3^ g∙mol^−1^.

Molar mass values in PS equivalent were converted in PP equivalent using the universal calibration equation [17]:

$${\mathrm{lnM}}_{\mathrm{PP}}=\frac{1}{1+\alpha_{\mathrm{PP}}}\cdot \ln\left(\frac{k_{\mathrm{PS}}}{k_{\mathrm{PP}}}\right)+\frac{1+\alpha_{\mathrm{PS}}}{1+\alpha_{\mathrm{PP}}}\cdot {\mathrm{lnM}}_{\mathrm{PS}}$$

where k_PS_, α_PS_, k_PP_ and α_PP_ are the Mark–Houwink constants for PS and PP. The following values were used [18,19]:

- PP: η_TCB, 135 °C_ = 16.1 × 10^−4^ · M_w_^0.733^;
- PS: η_TCB, 135 °C_ = 16.1 × 10^−4^ · M_w_^0.677^.

The different layers of the masks were analyzed in duplicate. The obtained uncertainties were quite small: the differences between the two analyses were lower than 5% for any given sample.

#### 2.3.3. Thermal-Desorption–Gas Chromatography–Mass Spectrometry (TD-GC-MS)

TD measurements were performed using a Perkin ELMER thermodesorber (TD 350) coupled to an Agilent gas chromatographer (GC-6890) that was connected with a mass spectrometer (MS5973N): the GC module allowed the separation of the volatile compounds and the mass spectrometry (MS) module enabled their identification. The materials were analyzed under the following conditions:

- Approximatively 10 mg of the surgery mask (one layer or four layers altogether) was introduced in a glass tube packed under helium atmosphere;
- Thermodesorption (TDS): 140 °C (10 min); helium 30 mL/min;
- Trapping: –30 °C; 40 °C/s to 180 °C; helium 30 mL/min;
- GC: 50 °C; 15 °C/min to 220 °C; helium 1.0 mL/min;
- MS: Transfer line temperature—200 °C; electron impact ionization—70 eV; scan range—10–300 m/z;
- Gases were concentrated on a cold trap and desorbed by a sudden rise in temperature. Only 2 vol% of the desorbed gases was sent to the GC-MS.

The entire analytical procedure was controlled using MassLynx software (Waters, MA, USA).

## 3. Results and Discussion

### 3.1. Characterization at the Molecular Scale

**Figure 2:**
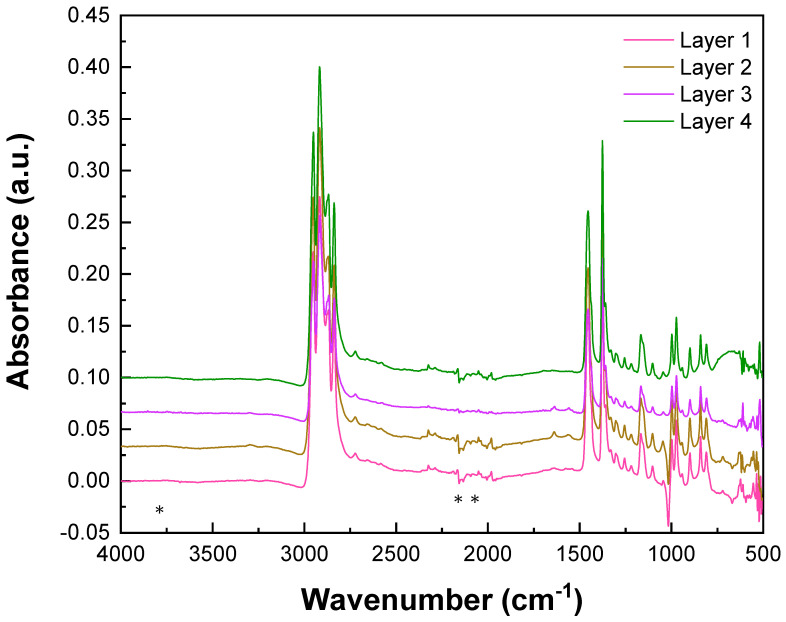
Infrared spectra of the four layers of the Valmy FFP2 mask. Stars are pointing bands that do not belong to the PP characteristic infrared spectrum. Stars correspond to bands observed only on layers 2 and 3 (for attribution, see Table 2).

The four layers of the Valmy FFP2 mask were analyzed by Fourier transform infrared spectroscopy in Attenuated Total Reflectance mode. According to their FTIR spectra, presented on Figure 2, the four layers corresponded to polypropylene fibers [20,21,22].

On layers 2 and 3, some additional bands, centered at 3295 cm^−1^, 1640 cm^−1^ and 1560 cm^−1^, were observed. They clearly indicate the presence of an additional molecule, which probably belongs to the amide family [23]. This is well ascertained in Table 2, which gives the amide II bonds’ characteristic positions along with the bands observed in the infrared spectra of layers 2 and 3 of the Valmy FFP2 mask, and also seems to be supported by the fact that this kind of molecule is known to be an effective process agent [24]. The bands at 1640 cm^1^ were slightly shifted compared theoretical values but we believe that the difference was due to the associated bond rather than the free one.

The additives identified in layers 2 and 3 were characteristic of molecules added to PP for the melt-blowing process. Moreover, it is known that FFP2 masks are generally composed of melt-blown and spun layers. With these two indications, the deduced composition was SMMS (spun/melt blown/melt blown/spun, from layer 1 to layer 4, respectively). These attributions are supported by the fact that the compositions of such masks are known to comprise multiple layers of polypropylene nonwoven fabrics, and that melt-blowing is a conventional fabrication method of micro- and nanofibers that is applicable for filtration [25].

**Figure 3:**
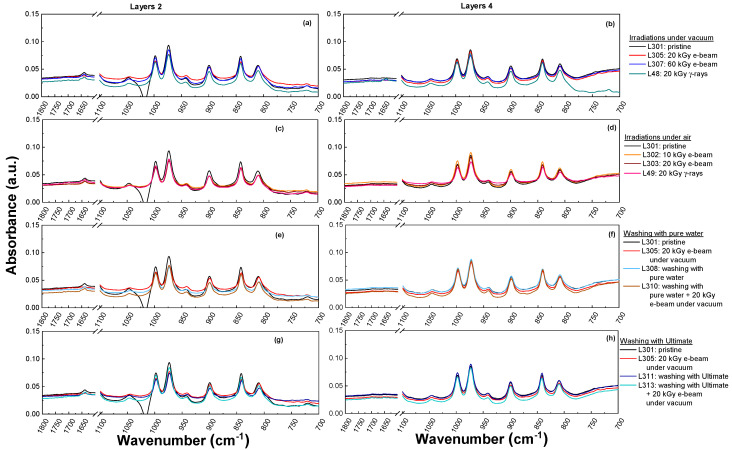
Infrared spectra of layer 2 and layer 4 of the Valmy FFP2 mask in ATR mode, before and after different sterilization protocols. Zoomed-in depictions of the 1800–1600 cm^−1^ (C=O area) and 1100–700 cm^−1^ (C=C area) areas. Line 1: irradiations under vacuum (**a**: Layer 1; **b**: Layer 3). Line 2: irradiations under air (**c**: Layer 1; **d**: Layer 3). Line 3: washing with pure water, followed by irradiation using e-beam under vacuum or not (**e**: Layer 1; **f**: Layer 3). Line 4: washing with Ultimate detergent, followed by irradiation using e-beam under vacuum or not (**g**: Layer 1; **h**: Layer 3).

After the different sterilization protocols, it appeared necessary to investigate the absence of significant evidence of PP-fiber degradation. Figure 3 displays zoomed-in depictions of the 1800–1600 cm^−1^ (carbonyls) and 1100–700 cm^−1^ (C=C double bonds) areas of the infrared spectra of two of the four layers of the FFP2 Valmy mask, that is, layer 2 (left column of Figure 3) and layer 4 (right column of Figure 3). Entire infrared spectra are presented in Figure S5 of the Supplementary Materials. The two other layers (layers 1 and 3) are not presented but the results are very similar (see Supplementary Materials—Figure S6 for the entire infrared spectra and Figure S7 for the zoomed-in depictions of the areas 1800–1600 cm^−1^ and 1100–700 cm^−1^). For all figures, changes in the chemical structures of the PP fibers are presented on the first line for irradiations performed under vacuum, on the second line for irradiations performed under air, on the third line for Valmy FFP2 masks that were washed with pure water prior to irradiation, and on the fourth and last line for masks that were washed with Ultimate detergent prior to irradiation.

It can be observed from Figure 3 that whatever the irradiation nature, the dose and/or the washing conditions prior to irradiation, the PP fibers of the different layers of the mask showed no evident modifications at the molecular level since neither the disappearance (for example, of C–H) nor the creation (for example, of the carbonyl groups) of an infrared band was observed.

Some bands showed slight changes upon the different sterilization protocols, which might have originated from the morphological evolution of the polymer chains. It is known from the literature [26,27] that changes in PP crystallinity can be evaluated using FTIR spectra via the following relation:

$$\chi_{C}=109\cdot \left(\frac{A_{997}-A_{917}}{A_{972}-A_{917}}\right)-31.4$$

where $A_{X}$ represents the absorbance of the band merging at the wavenumber X cm^−1^.

Hence, crystallinity changes in the different layers were evaluated. Corresponding values are gathered in Table S1 of the Supplementary Materials, while crystallinity values are given in Table 3 of Section 3.2.

### 3.2. Characterization at the Macromolecular Scale

Even in the absence of detection of trackers evidencing the degradation of PP fibers, it seemed interesting to quantify the damages at the macromolecular scale from the measurement of the average molar mass to ascertain the residual ductility of the FFP2 masks. It is actually known that PP embrittlement occurs at low oxidation level [28]. Table 3 gathers the average molar masses of the different layers sterilized masks along with the crystallinity ratio of the different layers that were obtained by FTIR analysis (from Table S1 of the Supplementary Materials) for each kind of sterilization condition. Layer 1, which is very thin, was hard to sample. Since it was not expected to play a significant role on the mechanical behavior of the sample, it was no longer investigated in terms of microstructural changes.

Let us recall that for PP, a critical molar mass value M’_C_ ~ 150 kg.mol^−1^ was proposed as end-of-life criterion corresponding to a strong loss of plasticity below this value [15]. Hence, in case of the analyses of the unsterilized mask (L301), it could be observed that layer 4 of both masks was made of “long PP chains” and was likely to display a plastic behavior. In principle, fibers should display no cracks at the surface, but SEM observations should be conducted to verify this [29]. This was not the case for the inner layers (layers 2 + 3), which had lower average weights and molecular weights, and thus, were expected to display more brittle behaviors.

Moreover, it can be noticed from Table 3 that e-beam irradiation under inert atmosphere led to modification of the length of the polymer chains, but the observed changes remained minor even for a 60 kGy dose. The presence of oxygen during e-beam irradiation led to the more severe drop of the average molar masses of the different layers of the FFP2 masks. However, it can be observed from Table 3 that the most important effect at the macromolecular scale was obtained for the γ-rays irradiation sterilization under vacuum and under air, the effect of the oxygen effect being even more marked in case of γ-ray irradiation sterilization. The molar mass decreased faster in the presence of oxygen than under vacuum/inert atmosphere.

The results for the L308 and L311 masks indicate that there was no—or almost no—effect of washing on FFP2 masks, whatever the washing conditions (i.e., with pure water, or with water and Ultimate detergent). The effect that was observed for the L310 and L313 masks seemed to be more linked to the irradiation process (L305) than to washing. Hence, it could be deduced from Table 3 that PP masks sterilized by washing were expected to keep their mechanical resistance. More precisely, it is possible that irradiation under air followed by washing could be much more detrimental to mechanical properties due to the leaching of short chains produced by chain scission reactions, and maybe because of the stronger surface oxidation, the latter being associated with surface cracking [29]. It was nonetheless observed in the literature that such effects associated with water can be observed at very long ageing times [30,31], whereas shorter ageing durations in water (1 to 4 h at 60 °C in solution of NaOH 10% at pH 14) were found to induce almost no effect [32].

Moreover, Table 3 recalls the crystallinities obtained from the infrared spectra. The analysis of the crystallinity evolution indicated that all the treatments seemed to slightly increase the crystallinity of the fibers, which might have been a consequence of the chain scission events evidenced here by GPC. This can be interpreted as follows: PP is in the rubbery state in the experimental conditions used for sterilization, and short chain segments can join the crystalline phase [15]. As slight as it may be, this evolution is an indication of the degradation of the FFP2 Valmy masks.

**Scheme 1:**
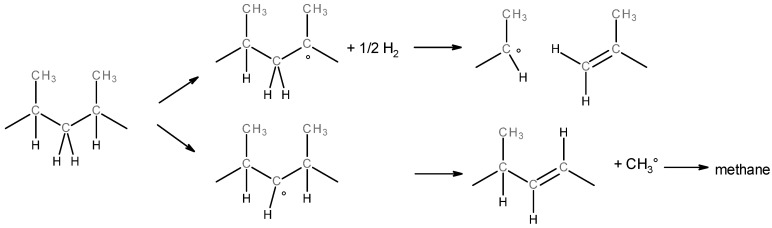
Mechanism of formation of *trans*-vinylene, hydrogen and methane from a radiolyzed PP [33,34].

To explain the observed trends, we can recall that when PP is irradiated under an inert atmosphere, the main defects formed include the formation of double bonds (*trans*-vinylenes, polyenes) and the release of gases (principally hydrogen and methane), as depicted in Scheme 1, together with scission and crosslinking [33,34].

The balance between chain scission and crosslinking is given as [35]:

$$\frac{1}{M_{n}}-\frac{1}{M_{n0}}=S-X\;\frac{1}{M_{w}}-\frac{1}{M_{w0}}=\frac{S}{2}-2X$$

Hence, with I being the dose rate, the scission and crosslinking yields can be evaluated using the chain scission and crosslinking concentrations by means of the following equations:

$$\frac{dS}{dt}=G_{S}\cdot I\;\frac{dX}{dt}=G_{X}\cdot I$$

In the case of e-beam irradiation (20, 60 kGy), the slope of the chain scission and crosslinking versus dose gave, respectively:

- Layers 2 + 3: G_S_ = 0.36 × 10^−7^ mol J^−1^ and G_X_ = 0.15 × 10^−7^ mol J^−1^;
- Layer 4: G_S_ = 0.42 × 10^−7^ mol J^−1^ and G_X_ = 0.05 × 10^−7^ mol J^−1^.

Those values are in acceptable agreement with the data given in the literature [36,37] where, for isotactic PP, the G_S_ values range from 0.24 × 10^−7^ mol J^−1^ to 0.25 × 10^−7^ mol J^−1^ and the G_X_ values range from 0.16 × 10^−7^ mol J^−1^ to 0.17 × 10^−7^ mol J^−1^. Unfortunately, for the other irradiation conditions, the data are too scarce for a reliable systematic assessment of the chemical yields obtained via chain scission and the crosslinking radiation.

**Scheme 2:**
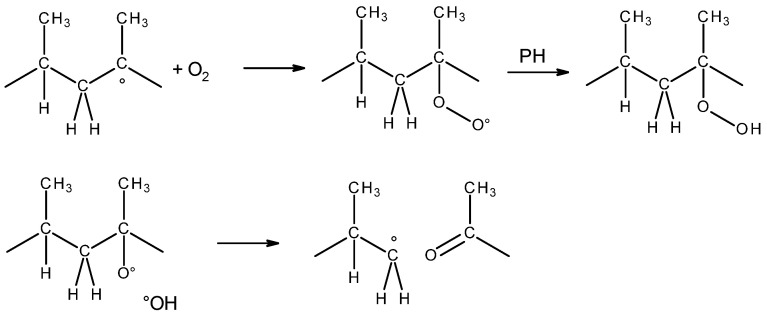
Mechanism of the formation of hydroperoxides and ketones from a radio-oxidized PP [38].

When PP was irradiated under homogeneous oxidative atmosphere, the main defects at the molecular scale were carbonyl-like bonds (ketones, carboxylic acids, and esters) and alcohol-like bonds (hydroperoxides and alcohols), as illustrated on Scheme 2 [38], together with the release of gases (hydrogen and methane, but also carbon monoxide and dioxide). Therefore, it was not surprising that the molar mass dropped faster for irradiation under air than under nitrogen.

### 3.3. Characterization of the Volatiles Trapped in the Mask

A quantification of the hydrogen and methane that evolved from the different layers of the FFP2 mask would have been interesting from a fundamental point of view, for the purpose of discussion in relation to the values of the radiochemical yields determined in the previous section. Here, we considered the evaluation of the trapped gases to be more important; these trapped gases could be identified by TD-GC-MS.

**Figure 4:**
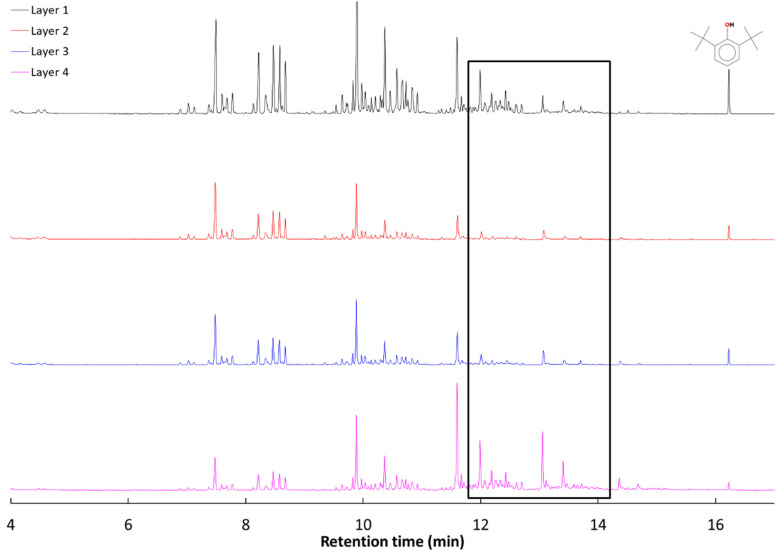
Chromatogram obtained by TD-GC-MS of the molecules trapped in the four layers of the Valmy FFP2 medical mask.

To identify molecules trapped in the different layers of the mask, an overall characterization of the layers was undertaken via thermal desorption. Chromatograms are presented on Figure 4. In the four layers, a massif was observed roughly between 7 and 14 min and was attributed, using the NIST database, to linear and branched alkanes, which probably came from the synthesis process. It was additionally observed that the heavy alkanes were most lacking in layers 2 and 3, i.e., in the melt-blown layers compared to the spun-bonded ones (see the box between 12 and 13 min in Figure 4). Finally, a peak characteristic of the butylated hydroxybenzene was found at 16.2 min for the four layers. This seemed to be a fragment of a stabilizer belonging to the hindered phenol family.

**Figure 5:**
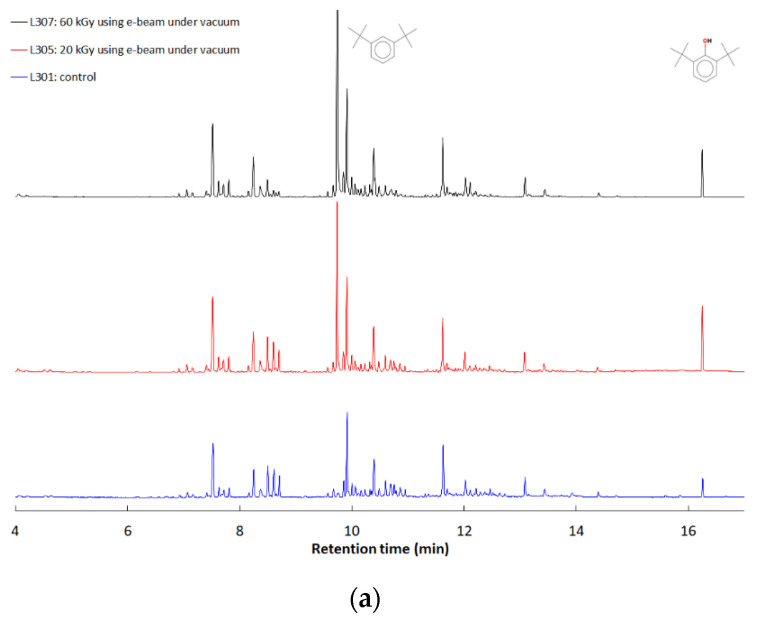

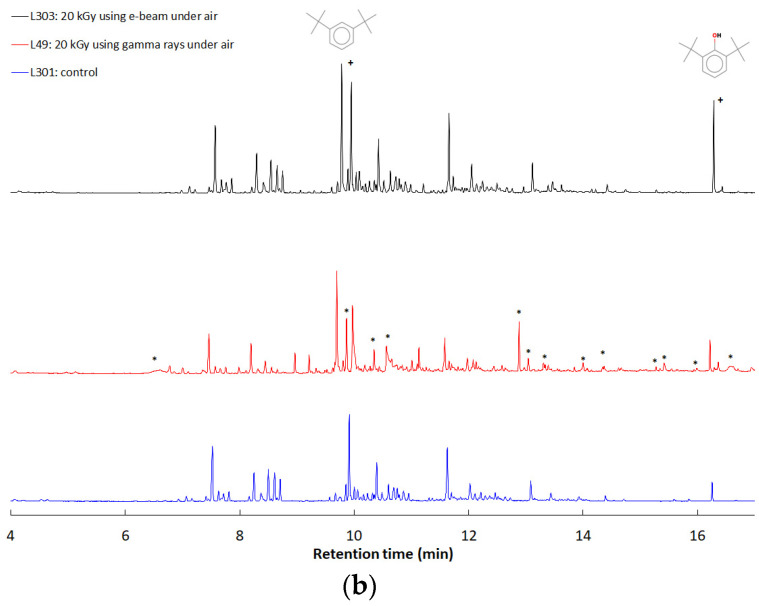
TD-GC-MS chromatograms of all four layers of the Valmy FFP2 mask under different conditions: (**a**) irradiated using e-beam under vacuum and (**b**) irradiated under air using an e-beam and under γ-rays.

Figure 5 displays the chromatograms obtained using thermal desorption for the Valmy FFP2 medical mask for all four of the layers, before and after irradiation. It was not possible to identify all the molecules present in the masks before and after irradiation; for this reason, only the main peaks that were present are analyzed in this section.

Characteristic peaks of fragments of a stabilizer of the hindered phenol family were identified: they are marked in Figure 5b with crosses, but they can also be found in all the TD-GC-MS chromatograms. For all of these molecules, which were released in small quantities, the sterilization conditions seemed to have no marked effect.

In case of irradiations performed under vacuum (Figure 5a), no modification of the chromatograms could be identified, indicating weak modification of the PP fibers under these irradiation conditions (even at 60 kGy). The polypropylene chain modifications were more obvious when irradiations were performed using γ-rays under air: the oxidation products are shown in Figure 5b (where they are marked with stars). Even under air, these degradation products were not observed under e-beam irradiation. This provides a clear indication that irradiations conducted under air, but performed at high dose rates of e-beam irradiation, are roughly equivalent to irradiations conducted under inert atmosphere.

When the FFP2 mask was washed prior to sterilization by irradiation, there was no evident modification of the trapped molecules, indicating that this preliminary step did not degrade the PP fibers. This observation was not only valid when pure water was used, but also when Ultimate detergent was used (see Figure S8 of the Supplementary Materials).

## 4. Conclusions

In the context of the SARS-CoV2 pandemic, it appears that studies on the resistance of surgical and FFP2 masks to sterilization processes were of primary necessity. As sterilization using irradiation (γ-rays and e-beam irradiations) is commonly used in the medical field, this process was tested for the first time. For acceptance purposes, washing prior to the sterilization process was mandatory; therefore, this preliminary step was also assessed, at the molecular and macromolecular scales, using PP fibers.

It was observed that washing prior to irradiation does not lead to modification of the polypropylene that constitutes the different layers of the Valmy FFP2 mask, be it using pure water or using a detergent (Ultimate detergent in our case). When irradiation was performed under vacuum, slight changes of the layers were observed. They mainly evidenced an increase in the crystallinity ratio and a decrease in the lengths of the chains upon irradiation. When using e-beams under oxidative atmosphere, the dose rate was found to be so important that modifications of the polymer were roughly equivalent to those under vacuum. The deepest modification of the different layers of the FFP2 mask was observed when irradiation was performed using γ-rays under atmospheric air. Under these conditions, the dose rate was sufficiently low that a homogeneous—or nearly homogeneous—radio-oxidation process could be observed, leading to the emission of oxidized gases, with part of them being trapped in the layers of the FFP2 masks. Additionally, under these conditions, crystallinity increased and the chain lengths decreased. The forthcoming conclusion should be ascertained by means of filtering experiments, but even if the modifications that were evidenced seem relatively weak, it is probable that they were sufficient to prevent the proper protection of the wearer after the sterilization process.

Since FFP2 mask materials are electrostatically charged to confer the necessary filtration level, a washing step will undoubtedly withdraw these surface charges. In this case, it is not the materials’ modification that will hinder the FFP2 masks’ reuse, but their filtration efficiency. A study on the evolution of the filtering properties should be conducted to complement the molecular- and macromolecular-scale modifications, realized in this study, of the PP fibers that are constitutive of the layers of the masks.

## Figures and Tables

**Table 1:** Sterilization conditions of the Valmy FFP2 medical mask. NB: L301 corresponds to unsterilized unwashed mask.

Sample Number	Sterilization Treatment	Target Dose (kGy)	Controlled Dose (kGy)	Dosimetric Uncertainty (%)	Irradiator
L301	Reference	0	-		IONISOS
L302	e-beam under air	10	10.4	4.6	
L303	e-beam under air	20	20.2	4.6	
L305	e-beam under vacuum	20	20.2	4.6	
L307	e-beam under vacuum	60	59.1	4.6	
L308	Washed with pure water	0	-		
L310	Washed with pure water + e-beam under vacuum	20	19.7	4.6	
L311	Washed with detergent (Ultimate)	0	-	4.6	
L313	Washed with detergent (Ultimate) + e-beam under vacuum	20	19.7	4.6	
L49	γ-rays under air	20	20.1	10	ArcNucleart
L48	γ-rays under vacuum	20	20.1	10	

**Table 2:** Attribution of the additional bands observed in the infrared spectra of layers 2 and 3 of the Valmy FFP2 mask (Lifshutz, 1997). ν: stretching; δ: bending.

Absorption Area (cm^−1^)	Amide II Bonds’ Attribution	Bands Observed on Layers 2 and 3 of the Valmy FFP2 Mask
3300–3050	υ(NH)—NH-associated bond	3295
1710–1660	υ(C=O)—C = O free bond	1640
1570–1515	δ(NH)	1560

**Table 3:** Average molar masses (in kg mol^−1^) and crystallinity ratios (in %) of the different layers sterilized masks for various sterilization protocols.

Sample Number	Sterilization Treatment	Target Dose (kGy)	Layers 2 + 3			Layer 4		
			M_w_	M_n_	χ_c_	M_w_	M_n_	Χ_c_
L301	Reference	0	94.9–101.6	33.0–28.2	26.85	251.4–251.5	71.5–71.9	23.8
L302	e-beam under air	10			32.7			29.5
L303	e-beam under air	20	71.4–107.9	26.7–28.1	30.5	154.0–140.0	50.8–48.1	26.6
L305	e-beam under vacuum	20	88.5–105.1	27.0–28.9	26.7	166.5–170.7	49.8–54.9	27.1
L307	e-beam under vacuum	60	112.1–90.7	23.3–31.3	30.6	155.9–156.6	49.2–47.9	23.8
L308	Washed with pure water	0	91.9–95.9	24.0–29.2	32.3	235.0–242.1	69.0–70.2	26.7
L310	Washed with pure water + e-beam under vacuum	20	90.2–98.6	24.0–25.9	33.1	172.3–182.6	54.9–64.9	33.1
L311	Washed with detergent (Ultimate)	0	74.4–81.6	20.5–27.6	32.2	246.8	67.8–74.7	30.1
L313	Washed with detergent (Ultimate) + e-beam under vacuum	20	80.2–82.3	23.0–24.0	30.8	180.4–186.0	55.4–56.0	26.8
L49	γ-rays under air	20	46.0–43.6	15.1–15.4	31.8	64.0–64.8	22.6	30.3
L48	γ-rays under vacuum	20	76.4–80.8	21.9–22.6	30.8	159.9–161.8	49.3–52.2	25.8

## Supplementary Materials

The following are available online at https://www.mdpi.com/article/10.3390/polym13234107/s1. Figure S1. Infrared spectra of the elastic strap of the Valmy FFP2 mask; Figure S2. Chromatogram obtained by TD-GC-MS of the molecules trapped in the elastic strap of the Valmy FFP2 medical mask; Figure S3. Infrared spectra of the elastic strap of the Valmy FFP2 mask before and after different sterilization protocols; Figure S4. TD-GC-MS chromatograms of the elastic strap of the Valmy FFP2 mask under different conditions; Figure S5. Infrared spectra of the Valmy FFP2 mask before and after different sterilization protocols; Figure S6. Infrared spectra of the Valmy FFP2 mask in ATR mode before and after different sterilization protocols; Figure S7. Infrared spectra of layer 1 and layer 3 of the Valmy FFP2 mask in ATR mode, before and after different sterilization protocols; Table S1. Crystallinities evolutions as a function of the layer under consideration and of the sterilization protocol process applied; Figure S8. TD-GC-MS chromatograms of the four layers altogether of the Valmy FFP2 mask under different conditions.
