# Supplementary material for: Sterilization Induced Changes in Polypropylene-Based Ffp2 Masks
Source: Polymers (Basel). 2021 Nov 25;13(23):4107. doi: 10.3390/polym13234107 (PMC8659121; doi:10.3390/polym13234107)
Supplement: Supplementary file 1 [file polymers-13-04107-s001.zip › polymers-1419277-supplementary.pdf]

polymers

Supplementary

Sterilization Induced Changes in Polypropylene-Based Ffp2 Masks

Emmanuel Richaud [1,*], Muriel Ferry [2], Floriane Carpentier [2], Sophie Rouif [3], Laurent Cortella [4] and Stéphane Esnouf [2]

1 PIMM UMR 8006, Arts et Metiers ParisTech, CNRS, CNAM, 151 bd de l'Hopital, 75013 Paris, France
2 CEA, Service d'Etude du Comportement des Radionucléides, Université Paris-Saclay, 91191 Gif-sur-Yvette, France; MURIEL.FERRY@cea.fr (M.F.); Floriane.CARPENTIER@cea.fr (F.C.); Stephane.ESNOUF@cea.fr (S.E.)
3 Ionisos SAS, 13 chemin du Pontet, 69380 Civrieux-d'Azergues, France; Sophie.Rouif@ionisos.com
4 ArcNucleart, CEA Grenoble, 17 avenue des Martyrs, CEDEX 9, 38054 Grenoble, France; laurent.cortella@cea.fr
* Correspondence: emmanuel.richaud@ensam.eu

Citation: Richaud, E.; Ferry, M.; Carpentier, F.; Rouif, S.; Cortella, L. Sterilization Induced Changes in Polypropylene-Based Ffp2 Masks. *Polymers* **2021**, *13*, 4107. https://doi.org/10.3390/polym13234107

Academic Editor(s): Alina Adams

Received: 28 September 2021
Accepted: 15 November 2021
Published: 25 November 2021

Publisher's Note: MDPI stays neutral with regard to jurisdictional claims in published maps and institutional affiliations.

Copyright: © 2021 by the authors. Submitted for possible open access publication under the terms and conditions of the Creative Commons Attribution (CC BY) license (https://creativecommons.org/licenses/by/4.0/).

1. Results on the elastic of the Valmy FFP2 mask

1.1. Characterization of the pristine FFP2 mask

The elastic strap of the Valmy FFP2 mask was analyzed by FTIR in ATR mode and according to the obtained spectrum (see Figure S1), this part of the mask is made of polyester. In the same way, Figure S2 presents the chromatogram of the elastic strap of the Valmy FFP2 medical mask. A very broad distribution of alkanes is observed between 8 and 20 min. The peak characteristic of butylated hydroxybenzene at 16.2 min is observed, along with peaks at 2.3 min and at 10.0 min, characteristic of 2-methyl-1,3-dioxolane and of acetic acid, respectively. Finally peaks characteristic of S-containing molecules (CS_2 and 2-(methylthio)benzothiazole, respectively) are observed at 1.5 min and at 17.2 min. Due to the function of the elastic strap, these molecules are very probably coming from a vulcanization process.

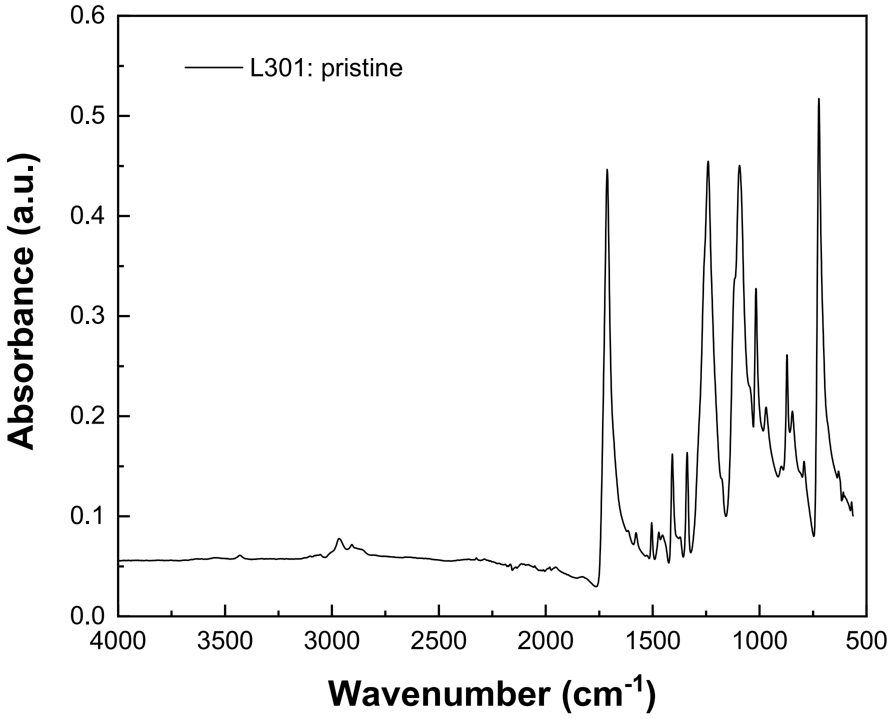

Figure S1. Infrared spectra of the elastic strap of the Valmy FFP2 mask.

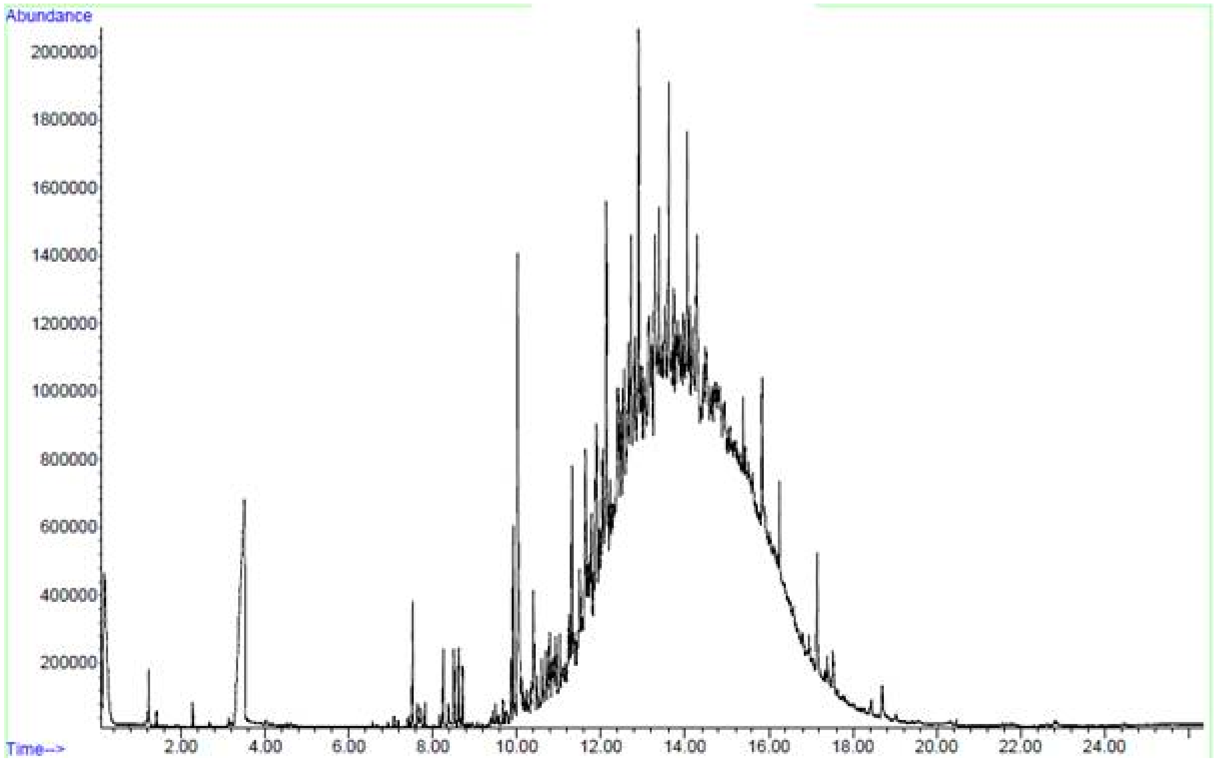

Figure S2. Chromatogram obtained by TD-GC-MS of the molecules trapped in the elastic strap of the Valmy FFP2 medical mask.

1.2. Characterization of the FFP2 mask after sterilization treatments

Figure S3 represents the elastic straps infrared spectra obtained after the different sterilization processes. No obvious modification could be evidenced whatever the sterilization protocol used.

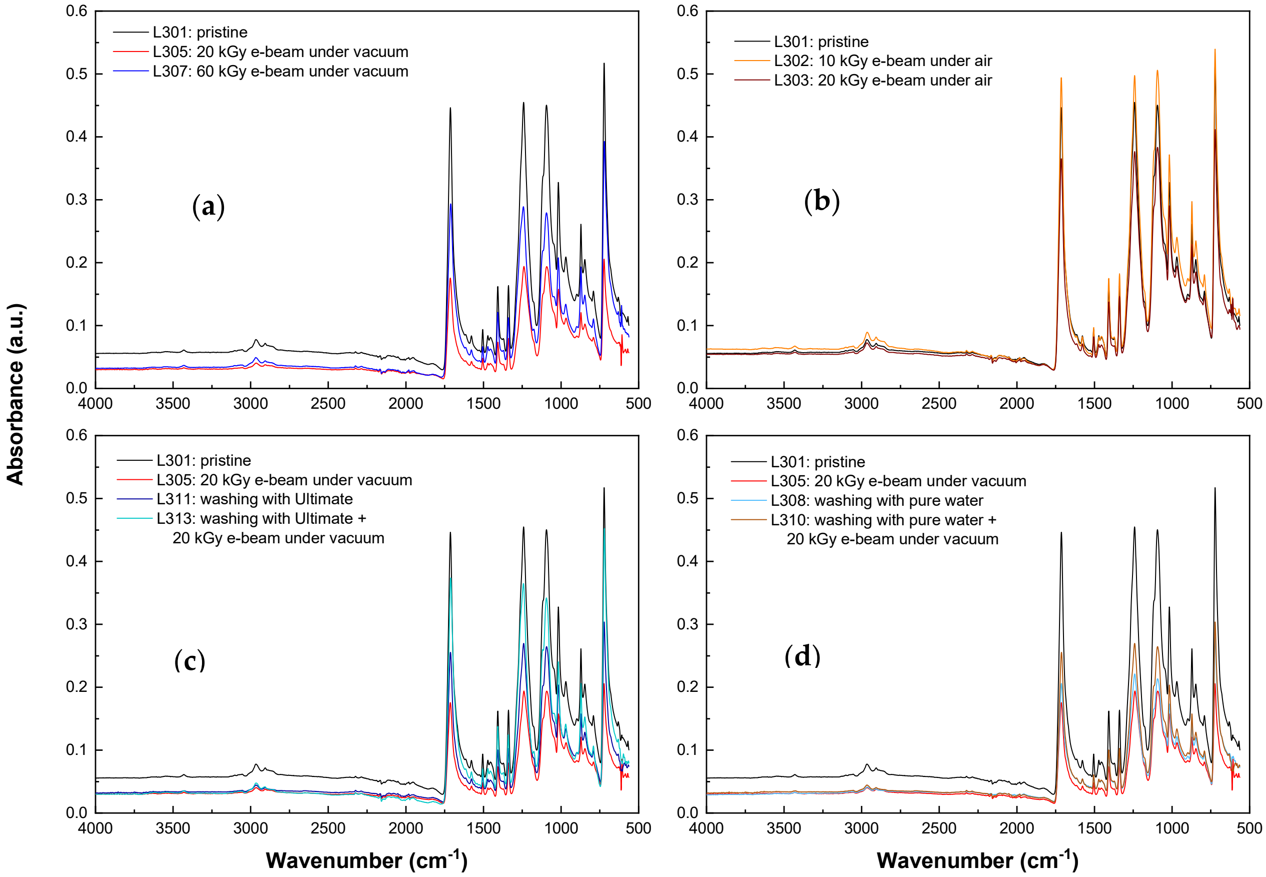

Figure S3. Infrared spectra of the elastic strap of the Valmy FFP2 mask before and after different sterilization protocols. (**a**) Irradiations under vacuum. (**b**) Irradiations under air. (**c**) Washing with pure water - and then irradiated using e-beam under vacuum or not. (**d**) Washing with Ultimate - and then irradiated using e-beam under vacuum or not.

Figure S4 presents the chromatograms for the elastic straps obtained using thermal desorption for the Valmy FFP2 medical mask. Peaks characteristic of fragments of a stabilizer of the hindered phenol family are evidenced, along with benzothiazole (14.2 min) and n-hexadecanoic acid (23.8 min). For all these molecules released in small quantities, the nature of the irradiation seems to have no marked effect: whatever the chosen sterilization protocol, S-containing degradation product molecules are identified.

The strap degradation is evidenced using TD-GC-MS: the strap will probably be the weakest part of the FFP2 Valmy mask.

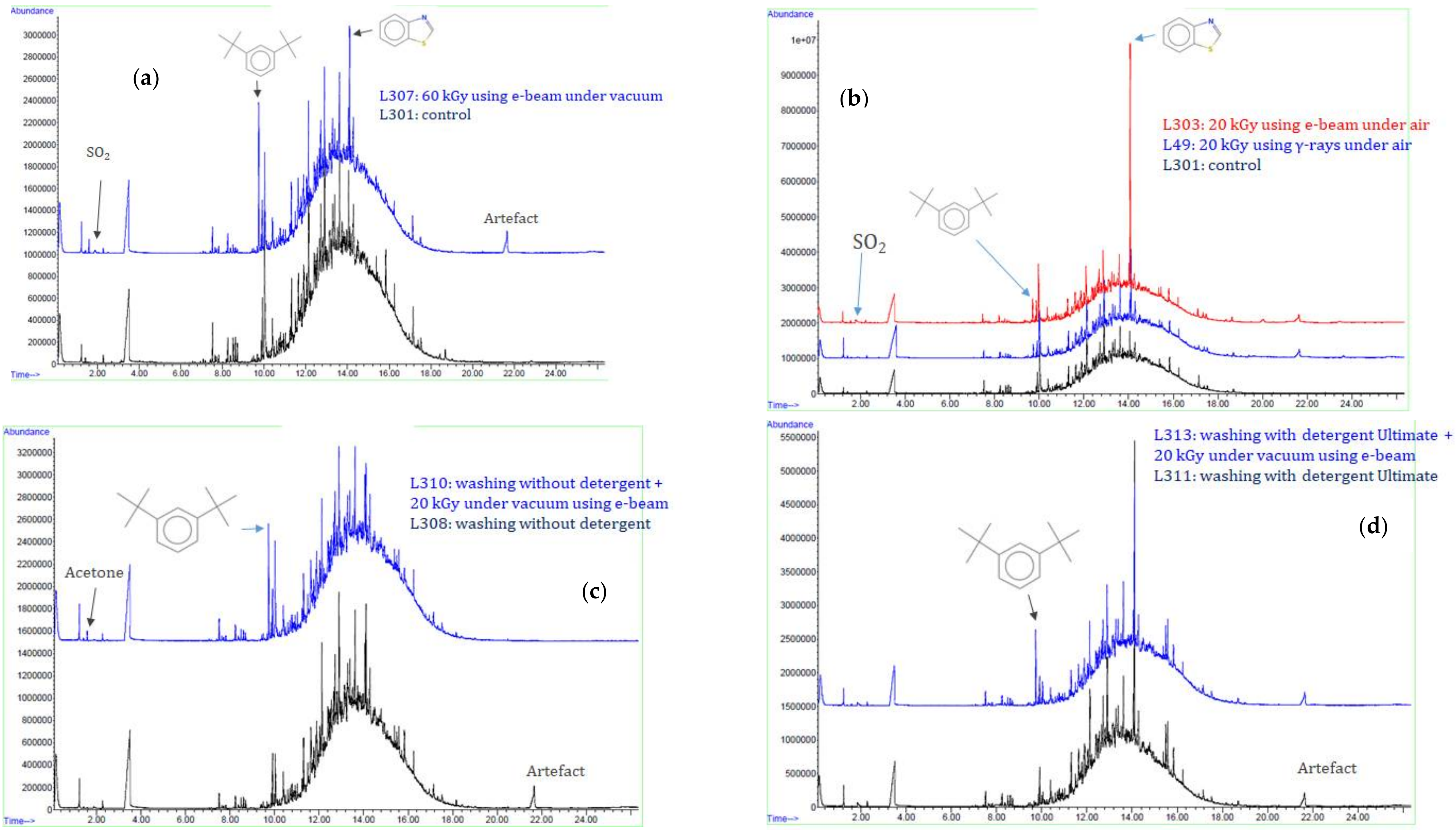

Figure S4. TD-GC-MS chromatograms of the elastic strap of the Valmy FFP2 mask under different conditions: (**a**) irradiated using e-beam under vacuum, (**b**) irradiated under air using e-beam and under γ-rays, (**c**) washed with pure water and then irradiated using e-beam under vacuum and (**d**) washed with Ultimate and then irradiated using e-beam under vacuum.

2. Complementary FTIR results on the four layers of the FFP2 mask

2.1. Infrared spectra of layer 2 and Layer 4 of the Valmy FFP2 mask

Figure S5 presents the layer 2 and layer 4 infrared spectra of the FFP2 mask in ATR mode.

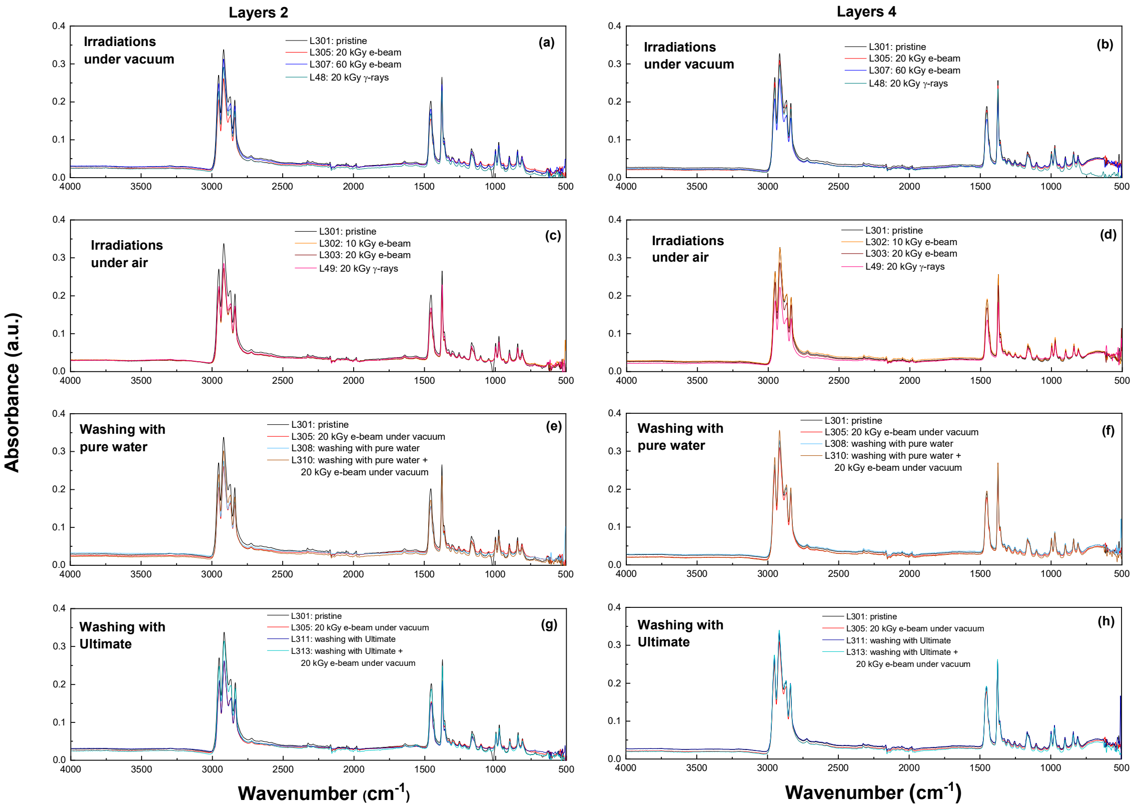

Figure S5. Infrared spectra of the Valmy FFP2 mask before and after different sterilization protocols. Line 1: Irradiations under vacuum (**a**: Layer 2 and **b**: Layer 4). Line 2: irradiations under air (**c**: Layer 2 and **d**: Layer 4). Line 3: Washing with pure water - and then irradiated using e-beam under vacuum or not - (**e**: Layer 2 and **f**: Layer 4). Line 4: Washing with Ultimate - and then irradiated using e-beam under vacuum or not - (**g**: Layer 2 and **h**: Layer 4).

2.2. Infrared spectra of layer 1 and Layer 3 of the Valmy FFP2 mask

Figure S6 presents the layer 1 and layer 3 infrared spectra of the FFP2 mask in ATR mode; Figure S7 presents, for these layers, the zooms in the areas 1800 - 1600 cm^{-1} (C=O area) and 1100 - 700 cm^{-1} (C=C area) of the infrared spectra of the FFP2 mask in ATR mode.

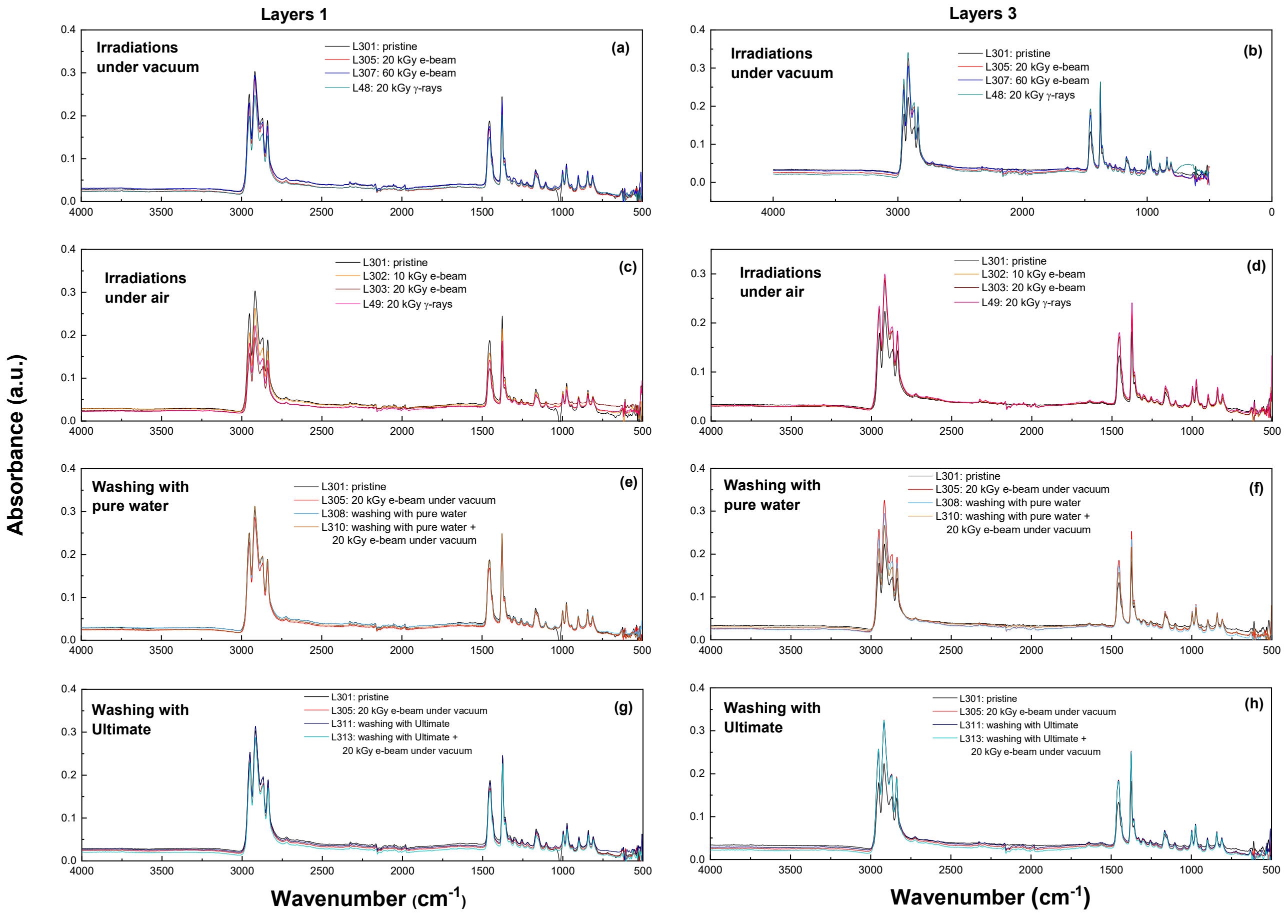

Figure S6. Infrared spectra of the Valmy FFP2 mask in ATR mode before and after different sterilization protocols. Line 1: Irradiations under vacuum (**a**: Layer 1 and **b**: Layer 3). Line 2: irradiations under air (**c**: Layer 1 and **d**: Layer 3). Line 3: Washing with pure water - and then irradiated using e-beam under vacuum or not - (**e**: Layer 1 and **f**: Layer 3). Line 4: Washing with Ultimate - and then irradiated using e-beam under vacuum or not - (**g**: Layer 1 and h: Layer 3).

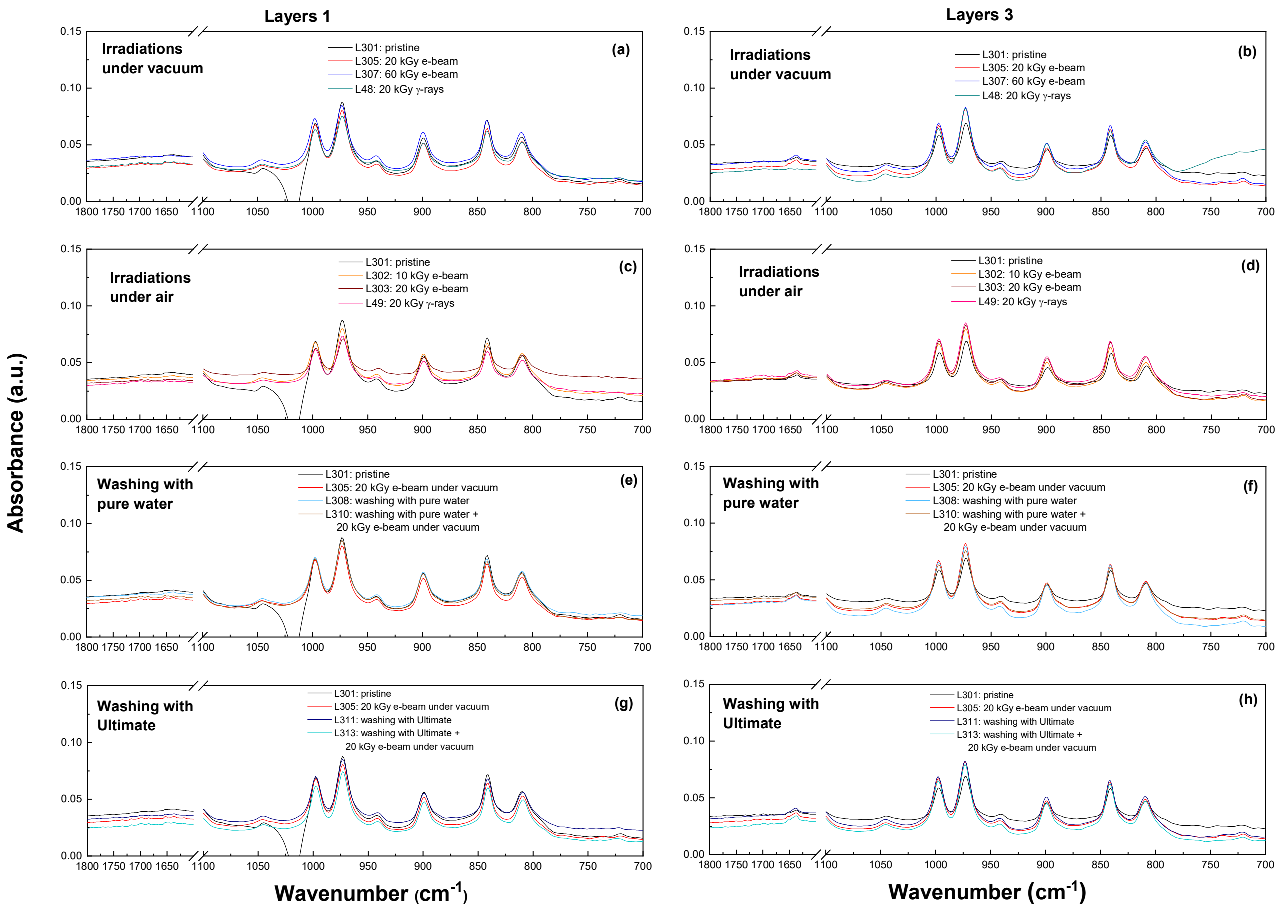

Figure S7. Infrared spectra of layer 1 and layer 3 of the Valmy FFP2 mask in ATR mode, before and after different sterilization protocols. Zooms in the areas 1800 - 1600 cm^{-1} (C=O area) and 1100 - 700 cm^{-1} (C=C area). Line 1: Irradiations under vacuum (**a**: Layer 1 and **b**: Layer 3). Line 2: irradiations under air (**c**: Layer 1 and **d**: Layer 3). Line 3: Washing with pure water - and then irradiated using e-beam under vacuum or not - (**e**: Layer 1 and **f**: Layer 3). Line 4: Washing with Ultimate - and then irradiated using e-beam under vacuum or not - (**g**: Layer 1 and **h**: Layer 3).

2.3. Crystallinity evolutions determined using FTIR

Table S1 gathers the values of the crystallinity percentages evolutions obtained as a function of the layer under consideration and of the sterilization protocol process applied.

**Sample number**	**Sterilization treatment**	**Dose (kGy)**	**Layer number**	**A997**	**A972**	**A917**	**%Crystal-linity**
L301	Pristine	0	1	0.0440	0.0630	0.0280	18.4
			2	0.0520	0.0710	0.0320	24.5
			3	0.0284	0.0388	0.0154	29.2
			4	0.0425	0.0586	0.0260	23.8
L49	γ-rays under air	20	1	0.0300	0.0422	0.0190	20.3
			2	0.0414	0.0546	0.0232	31.8
			3	0.0420	0.0564	0.0246	28.2
			4	0.0288	0.0390	0.0155	30.3
L48	γ-rays under vacuum	20	1	0.0358	0.0477	0.0225	26.1
			2	0.0456	0.0596	0.0270	30.8
			3	0.0462	0.0638	0.0292	22.2
			4	0.0467	0.0617	0.0277	29.5
L302	e-beam under air	10	1	0.0375	0.0496	0.0256	22.6
			2	0.0371	0.0491	0.0200	32.7
			3	0.0413	0.0547	0.0234	30.9
			4	0.0470	0.0621	0.0279	29.5
L303	e-beam under air	20	1	0.0230	0.0310	0.0139	26.6
			2	0.0377	0.0502	0.0213	30.5
			3	0.0432	0.0575	0.0260	28.1
			4	0.0370	0.0509	0.0212	26.6
L305	e-beam under vacuum	20	1	0.0432	0.0562	0.0265	29.9
			2	0.0336	0.0459	0.0218	22.0
			3	0.0451	0.0605	0.0242	31.4
			4	0.0425	0.0578	0.0248	27.1
L307	e-beam under vacuum	60	1	0.0434	0.0552	0.0298	27.0
			2	0.0459	0.0607	0.0269	29.9
			3	0.0441	0.0581	0.0251	31.4
			4	0.0350	0.0474	0.0223	23.8
L308	Washed with pure water	0	1	0.0432	0.0582	0.0277	24.0
			2	0.0352	0.0471	0.0180	33.0
			3	0.0401	0.0530	0.0224	31.6
			4	0.0457	0.0618	0.0273	26.7
L310	Washed with pure water + e-	20	1	0.0440	0.0601	0.0282	22.6
			2	0.0437	0.0574	0.0243	32.5

	beam under vacuum		3	0.0480	0.0625	0.0264	33.8
			4	0.0517	0.0662	0.0307	33.1
L311	Washed with detergent (Ultimate)	0	1	0.0402	0.0556	0.0235	25.3
			2	0.0331	0.0441	0.0168	33.7
			3	0.0451	0.0592	0.0264	30.7
			4	0.0483	0.0636	0.0285	30.1
L313	Washed with detergent (Ultimate) + e-beam under vacuum	20	1	0.0394	0.0521	0.0239	28.5
			2	0.0493	0.0639	0.0283	32.9
			3	0.0458	0.0625	0.0252	28.8
			4	0.0477	0.0644	0.0286	26.8

Table S1. Crystallinities evolutions as a function of the layer under consideration and of the sterilization protocol process applied.

2.1. Identification of the different peaks observed using TD-GC-MS

Figure S8 presents the chromatograms obtained using thermal desorption for the Valmy FFP2 medical mask for the four layers altogether, before and after sterilization under different conditions: (a) washed with pure water - and then irradiated using e-beam under vacuum or not - and (b) washed with Ultimate - and then irradiated using e-beam under vacuum or not -.

It is believed that the loss of caprolactone but also the decrease of high molar mass alkanes are due to the washing process. In fact, a standard personal washing machine was used and the observed difference might be due to residues inside this household machine.

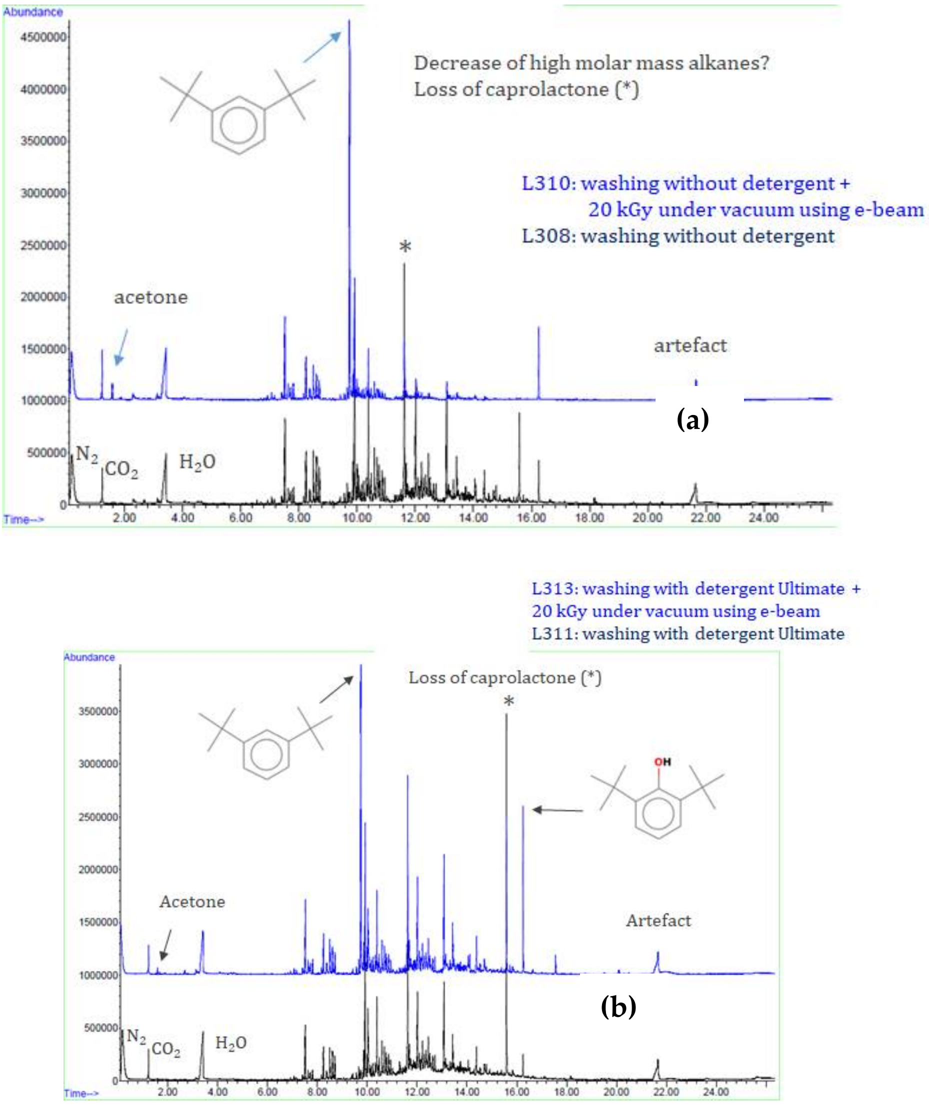

Figure S8. TD-GC-MS chromatograms of the four layers altogether of the Valmy FFP2 mask under different conditions: (**a**) washed with pure water - and then irradiated using e-beam under vacuum or not - and (**b**) washed with Ultimate - and then irradiated using e-beam under vacuum or not -.
